# The Mind-Brain Relationship as a Mathematical Problem

**DOI:** 10.1155/2013/261364

**Published:** 2013-04-14

**Authors:** Giorgio A. Ascoli

**Affiliations:** Krasnow Institute for Advanced Study, George Mason University, Fairfax, 22030-4444 VA, USA

## Abstract

This paper aims to frame certain fundamental aspects of the human mind (content and meaning of mental states) and foundational elements of brain computation (spatial and temporal patterns of neural activity) so as to enable at least in principle their integration within one and the same quantitative representation. Through the history of science, similar approaches have been instrumental to bridge other seemingly mysterious scientific phenomena, such as thermodynamics and statistical mechanics, optics and electromagnetism, or chemistry and quantum physics, among several other examples. Identifying the relevant levels of analysis is important to define proper mathematical formalisms for describing the brain and the mind, such that they could be mapped onto each other in order to explain their equivalence. Based on these premises, we overview the potential of neural connectivity to provide highly informative constraints on brain computational process. Moreover, we outline approaches for representing cognitive and emotional states geometrically with semantic maps. Next, we summarize leading theoretical framework that might serve as an explanatory bridge between neural connectivity and mental space. Furthermore, we discuss the implications of this framework for human communication and our view of reality. We conclude by analyzing the practical requirements to manage the necessary data for solving the mind-brain problem from this perspective.

## 1. Introduction

The relationship between mind and matter has been a fundamental topic of investigation in many if not all cultures and traditions since the most ancient records of human thought, from the Hindu orthodox school of Sankhya nearly 27 centuries ago [1] to the classic Greek philosophy of Plato (e.g., in the dialogue Phaedo) 300 years later [2]. With few exceptions (most noticeably that of panpsychism: [3]) most theories of the mind throughout history related it to the body and its various parts, including the heart in Aristotle's view [4] and the endocrine pineal gland in the work of Descartes [5]. Early physicians Hippocrates [6] and Galen [7] were among the first influential proponents of the central role of the brain in the operation of the mind based on anatomical and physiological observations. The development of modern neuroscience led to the (still ongoing) accumulation of massive evidence that irreversibly linked the mind to the brain [8].

The goal of this spotlight paper is emphatically *not* to provide an extensive review or even a balanced perspective of the enormous body of work on the brain-mind relationship in cognitive philosophy. To appreciate the breadth and depth of this topic, we refer the reader to a sample collection of over 200 articles on consciousness and neuroscience available online [9]. Instead, we selectively review a set of specific topics in neuroscience and cognitive science together supporting the notion that, within a particular interpretation, certain aspects of the mind-brain relationship can be framed as a rigorously defined and in principle soluble mathematical problem. In order to build this argument, we first describe in the next two sections the somewhat delimited facets of the mind and the brain we aim to bridge together. Next, we explicate what in our view would count as “solving” the mind-brain problem. Then we elaborate on relevant general topics in the investigation of the brain and of the mind that will likely play a central role in a satisfactory explanation of the mind-brain relationship. Finally, we overview a sample of more specific available frameworks in neuroscience and cognitive science that appear particularly promising among the known existing candidates to help crack the mystery of the link between mental and neural activities.

## 2. Declarative Mental States: Content and Meaning

The term “mind” is commonly employed to signify a broad variety of connotations even within the scientific discourse [10, 11]. When referring to the mind-brain relationship, mind is most often taken to stand for human consciousness [12]. Consciousness is itself challenging to define, which may be viewed as a puzzling paradox, considering that it constitutes perhaps the most immediately and intimately accessible characteristic of the life of every person. Consciousness cannot even be taken as a trivial or necessary feature that is always present in our existence, because we all experience the transition of fading into dreamless sleep and awaking from it tens of thousands of times in the course of a typical life span. The distinction between inner mental content and outer behavioral observables is equally apparent when considering the obvious difference between dreamless sleep and a vivid dream [13]. Judging from the paralyzed body (except for eye movement), an external spectator could not even begin to guess the mental state of a dreaming person [14]. In contrast, we often toss, turn, kick, twitch, mumble, and moan during dreamless sleep [15].

Here and in the rest of this paper we do not consider the legal or medical definitions of being conscious as opposed to unconscious, because the shared goal of physicians and lawmakers is to evaluate other people rather than themselves. Thus, they must necessarily take an objective, third-person perspective, that is, one entirely based on behavioral evidence. In contrast, what makes mental states so hard to relate to the brain, the body, and matter in general is precisely their being felt from “within”, subjectively, or from a first-person perspective [16]. Similarly, there are clear resemblances between the properties of consciousness and those of attention [17]. Neuroscience has advanced considerably in the elucidation of the neural mechanisms underlying attentive processes (e.g., [18]). Nevertheless, recent evidence clearly indicates a double dissociation between attention and consciousness [19].

Excluding the immediately cyclical definitions (such as equating consciousness to being aware and awareness to being conscious), one of the simplest approaches to describe consciousness is to define it as “what it feels like being” [20]. Thus, a particular conscious sensation (such as the taste of chocolate) or thought (such as remembering someone's name) would just correspond to what it feels like to taste chocolate or what it feels like to remember that person's name. The challenge of the mind-matter relation, then, is to understand how particular configurations of matter (e.g., a person's body or brain) or material processes (the dynamic activity of that body or brain) systematically coexist or cooccur with feeling particular mental states [21]. The ultimate “hard problem” is to explain why any material entity should feel like anything at all.

Many aspects of conscious experience can be reported explicitly by the subject [22, 23] either internally, by inner dialogue or memory rehearsal, or externally, such as by a verbal description or numerical rating. A defining characteristic of such *declarative content* of the conscious mind is that it can be communicated. The most common form of communication among human beings is through spoken or written language, but declarative content can also be communicated by other means, such as body gesture, drawings, movies, and many others. Independent of the signs or “signifiers” used (e.g., words and sentences in language), the declarative content they communicate is what we call their *meaning*. The field of semantics studies meaning and its communication by signifiers.

Consider a subject experiencing a certain declarative mental state (e.g., anger) and communicating it to another person “I'm mad at you!”. This message informs the recipient of the sender's conscious content. A fascinating facet of declarative communication is that the content intended by the sender is generally not identical to that understood by the recipient (e.g., why and how seriously is s/he angry?). We will return to this mismatch at the end of the paper.

## 3. The Brain: Spatial and Temporal Patterns of Neural Activity

Overwhelming scientific data unequivocally link the conscious mind to the brain, as opposed to matter at large. The general consensus on this interpretation in the research community boosted the popularity of the materialist credo that “the mind is what the brain does” [24]. Available evidence, however, is more specific than generically pointing to the brain as the most relevant organ of the body in relation to the mind. Particular regions of the brain are more directly and intimately involved with mental activity, including the thalamocortical system [25] and the hippocampal complex [26]. Other parts of the central nervous system, such as the cerebellum and the spinal cord, appear less pertinent to the declarative content of the mind. 

The majority opinion is that what renders the parts of the brain relating to the mind “special” is not the intrinsic substance they are made of but their functional organization. In fact, those same thalamocortical and hippocampal regions are definitely active even during dreamless sleep, but their activity is different from that during conscious states [27]. Thus, it is the information process, or computation, carried out by these brain structures, that most closely corresponds to consciousness [28, 29]. Implementing that same computation in different mediums, in artificial machines, or in virtual environments, would then in principle also result in conscious experience.

Unfortunately, however, what might in fact constitute the essence of this computation, or its neural implementation, is far less clear. Proposals range from involvement of broadly distributed oscillatory rhythms ([30]; see, however, [31]) to activation of highly specific “concept neurons” [32]. For the purpose of this paper, we do not need to dwell into specific hypotheses, except noting a general consensus supporting spatial-temporal patterns of neural activity as key material correlates of conscious experience. The carriers of neural activity are neurons, biological objects each occupying a physical volume in the brain. Thus, the complete electric and chemical record of every small portion of each neuron and their connections in the entirety of the appropriate brain networks throughout the course of an arbitrarily large number of declarative mental states would in principle include all the necessary information about “the brain” that is relevant to the content of the mind [33].

Within the particular framework we have chosen to adopt, the key players of the mind-brain relationship can be summarized as the following. On the one hand, each of us has access to the immediate experience of what it feels like to be, in any one moment of our conscious existence and throughout our life span. We can communicate that experience to each other, and our mental states are altered by receiving those messages. On the other hand, the brain is a dynamical information processing system, consisting of a large number of neurons, each characterized at any one time by the spatial distribution of its electrochemical state. Within a brain, neurons continuously exchange signals with each other, mutually affecting their spatially distributed electrochemical state.

In this framework, the mind-brain problem can be formulated as a simple question. Why and how do certain spatio-temporal patterns of neural dynamics relate to declarative mental states? In other words, why do those “neural dynamics that feel like something” feel like those (or any!) experiences?

## 4. What Would Count as an Explanation? 

Many relationships are routinely observed in the course of everyday life, such as how the weight of bodies relates to their size or how satisfying it feels to drink when thirsty. Why is the mind-brain relationship so different as to constitute a *problem*? The main challenge seems to be that typically related observables are somehow of the “same kind.” For example, size and weight are both physical attributes of material objects, while thirst and satisfaction are both subjective sensations of minds. Brains and minds are so different from each other as to inspire several kinds of dualist philosophies in the course of time. This fundamental difference has been considered challenging by scientists and philosophers alike, from confronting possible inconsistencies in temporal dynamics [34] through a retreat to the acceptance of limited explanations [35], to overt calls for giving up scientific accounts altogether [36].

The more extreme positions maintaining that there is no solution to the mind-brain problem have been refuted both on philosophical [37] and scientific grounds [38]. The key issue is in fact to understand what would count as a solution. In other words, if the mind-brain relationship can be explained, what type of explanation is being sought? To answer this central question, it is useful to consider previous scientific breakthroughs that resulted in satisfactory understanding of relationships that had been considered difficult to explain before. The most illustrative examples would concern discoveries relating (sets of) observations that appeared completely disparate from each other, linking phenomena seeming of “different kinds.”

A prime case in point is the explanation of thermodynamics in terms of statistical mechanics. The 1687 publication of Newton's Principia [39] consolidated a century of gains from the scientific method introduced by Galileo [40] into a coherent and complete theory of mechanics. Newton's famous laws relating force to acceleration and defining the mutual attraction of masses by gravity provided an excellent description of planetary motion as well as accurate predictions of interactions among material objects. More than one hundred years later the first comprehensive treaty on thermodynamics was published [41], describing the relationship between heat, energy, temperature, and what was later called entropy. 

Thermodynamics appeared completely independent of Newton's mechanics which explained instead how bodies move and respond to forces. While both mechanics and thermodynamics were (and still are) recognized as landmark advancements of scientific progress, they seemed to describe phenomena of different kinds. Yet, some relationships, discovered earlier by the likes of Gay-Lussac, Avogadro, and Boyle [42], consistently crossed that divide, such as the proportionality of gas pressure (a mechanical attribute) and temperature (a thermodynamic one) in a container of fixed volume. Moreover, simple observations remained challenging to reconcile on both sides of the divide, such as why matter can apparently be heated up to arbitrarily high temperatures but not cooled off below a certain limit (“absolute zero” or −273.15°C) and the continuously jittery (“Brownian”) motion of particles visualized under a microscope [43].

Between 1856 and 1871, Kroenig, Clausius, Maxwell, and Boltzmann developed a “kinetic theory” explaining thermodynamics in terms of mechanics, based on pioneering ideas originally proposed more than a century earlier [44]. Specifically, temperature relates to the average quadratic velocity of a large number of microscopic particles and pressure to their momentum. These relationships intuitively match the observation that, when warming up one's hands by rubbing them together, the faster the movement, the higher the generated heat. Entropy is related to the number of possible states a system can be in, which clarifies why disorder tends to increase in the absence of other constraints. The demonstration of the equivalence between thermodynamics and mechanics is as convincing and direct as the derivation of the corresponding thermodynamic and mechanical laws. In particular, the law of gas can be derived from Newton's formulas. Furthermore, this link solved the mystery of the lower bound of temperature: there is no practical limit to the maximum speed that particles can have, but they cannot go any slower than being perfectly still (corresponding to absolute zero). Notably, the kinetic theory also led to the famous 1905 Einsteinian explanation of Brownian motion (recounted in [45]).

Another striking example of scientific advancement linking phenomena “of different kinds” is the explanation of optics in terms of electromagnetism. The phenomena of refraction and diffraction are fully explained by Maxwell's equations of electrodynamics once light is understood as electromagnetic wave. Yet the properties of mirrors and the passage of light through various media (such as air and water) seem so different from the phenomena of electric current and magnetic dipoles. Other illustrations of the same relationships are the quantum physics foundation of chemistry, the genomics bases of genetics, and the explanation of neuronal firing in terms of voltage-dependent sodium and potassium channels [46].

When Mendeleev compiled the first draft of the Periodic Table of the Elements, there was no physical justification of the observed proportions of chemical reactions: the truth and correctness of the Table relied on its predictive power and descriptive elegance. Mendeleev's few initial “mistakes,” most of which he himself corrected in subsequent versions, were due to using the atomic mass (the observed “chemical weight” of a substance) rather than the atomic number (the number of protons in the nucleus) as the organizing principle. The atomic nucleus was discovered 42 years later, just 4 years after Mendeleev's death. Thus his corrections, required to describe then-available data parsimoniously, de facto predicted the atomic number nearly half a century prior to its actual discovery. The subsequent development of quantum mechanics showed that the atomic number emerged as a mathematical necessity from more fundamental assumptions, such as Schrödinger's wave equation or Heisenberg's uncertainty principle. Today we readily accept that many chemical phenomena (e.g., the tendency of metals and halogens to combine, as well as their “valence” ratios) are ultimately “explained” by quantum mechanics, even if these two phenomena would appear to relate to different and independent aspects of reality.

Is there a common thread in these seemingly disparate, if illustrious, precedents? We purport that the illusion of mystery at one level (e.g., the law of gases, light diffraction, and the combining ratios of chemical elements) evaporated as soon as those same sets of properties were discovered or demonstrated at a different level (particle kinematics, electromagnetic waves, and quantum orbitals). Breakthroughs of these sorts are fundamentally distinguishable from hand-waving “just-so” speculations because once the parallel is drawn, it systematically explains and predicts an entire body of observations rather than isolated phenomena. Here we surmise that the content and meaning of mental states, the most inescapable yet ineffable puzzle of human cognition, will eventually be understood as a direct reflection, if not simply an aspect, of brain computation, much like thermodynamics is statistical mechanics. Given the complexity of brain dynamics and of our inner experience, we expect the mathematical constructs connecting neuroscience and mind to be far less simple than Newton's, Maxwell's, or Schrödinger's equations. The principle of what would count as an explanation, however, remains the same: to solve the mind-brain conundrum one “only” needs to show that the math of the mind and the math of the brain are equivalent. 

We are not simply proposing mental properties to be probabilistically supervenient on brain properties, that is, that they can be inferred statistically from brain measures within any given error rate [47]. On the contrary, we are asserting the possibility of a formal equivalence between the two, through all temporal scales and plastic changes [48]. The explanatory power of mathematical theory in neuroscience is recognized in principle [49], but the extent of its reach has not yet been fully realized, and the path forward has never been chartered before. This is in stark contrast to the third simulation-based leg of scientific progress (complementary to experiments and theory), which is blossoming into maturity in computational neuroscience and cognitive modeling alike [50] and in the study of consciousness in particular [51, 52]. 

## 5. Neural Connectivity as the Most Informative Constraint in the Brain

To explain the equivalence of brain and mind by mapping them onto each other, it is essential to identify the relevant levels of analysis in order to define proper mathematical formalisms for their quantitative description. We start from the brain in this section and tackle the mind in the next. Nervous systems are gigantic networks of intercommunicating neurons. From the computational point of view, it matters relatively little that neurons are electrical devices. Instead, brain signal processing is fundamentally dependent on circuit connectivity [53]. Specifically, how neurons are connected to each other constrains network dynamics [54] and therefore determines the possible flow of information transmission [55]. 

A human brain has an estimated ~10^11^ neurons and 10^15^ synapses [56], which could in theory form a humongous number (~10^10^16^^) of distinct connectivity patterns, between a googol (10^10^) and a googolplex (10^10^100^^). Because real brains are wired to a certain degree according to stereotypical principles [57], the actual number of connectivity patterns that could be found in any one human brain is undoubtedly lower. However, brain circuitry is neither random nor regular, and the information content of a single human brain remains far greater than the number of fundamental particles in the whole universe, let alone just the complete biochemical specification of that individual brain. Thus network connectivity is necessarily more informative than the entire molecular profile of each of all of its neurons, including the expression of every gene and protein constituting the biophysical machinery at the basis of neuronal electrophysiology.

While neuroanatomy provides the foundational roadmap of information transmission in nervous systems, neural activity is itself characterized by chaotic dynamics [58] typical of complex systems [59, 60]. As these aspects are particularly relevant to conscious brain function [61], a full understanding of the brain as it relates to mental content will have to integrate adequate accounts of both neural dynamics and connectivity [62–64]. Nevertheless, the network architecture specification is absolutely central to the assumed correspondence between spatial-temporal patterns of neural spiking and mental states.

The notion of “connectomics”, characterizing the circuit blueprint of the nervous system [65], has progressively grown close to practical feasibility with the recent dramatic advancements in genetic manipulations allowing for multicolor microscopic visualization [66, 67]. Two distinct connectome concepts refer, respectively, to the cellular level, or “synaptome” [68, 69], and the regional level, or “projectome” [70]. The former is further distinguished in the dense reconstruction of the entire synaptic matrix and the statistical potential of synaptic connectivity, both highly relevant to computational processing [71–73]. In contrast, the much coarser description of regional connectivity has less direct implications for a mechanistic understanding of brain cognition. However, this latter approach is also substantially more realistic to achieve in the near future, using existing histological techniques in animal models [74, 75] or noninvasive imaging in humans [76, 77].

A number of “big-science” as well as grass-root data acquisition efforts are underway in both cellular and regional connectomics. These include the Human Connectome Project [78, 79], the 1000 Connectomes Project [80, 81], the Mouse Brain Architecture Project [82], and the FlyCircuit Database [83], among many others. This flurry of developments along with nonconventional approaches (e.g., [84]) is generating a wave of optimistic expectation in the research community that massive neural connectivity data will become increasingly available in the foreseeable future.

The branch of mathematics dealing with connectivity is graph theory. In light of the previous considerations, it is not surprising that graph theory has become a considerably popular topic in neuroscience (e.g., [85, 86]). It is remarkable that important properties of general graphs that have been found to apply to many types of networks, including random connections [87], small-world attributes [88], scale invariance [89], and motif distributions [90], are prominently relevant to neural circuits [91–94].

The application of graph theoretic analysis to neural circuit has already revealed a number of features, including network communities [95] and rich clubs [96], but also general principles of wiring economy [97] and network organization [98] as well as potential implications of circuit structure on signal communication [99, 100]. It is important to stress that, while two cells are never exactly alike, neurons can be organized in distinct classes such that neurons within each class are much more similar to each other than across classes [101]. Thus the statistical properties of brain connectivity are likely to be strongly determined at the level of connection probability among neuron classes. Initial progress is being made in the application of the relevant field of mathematics, stochastic block modeling, to this problem [102, 103].

Two further facets are worth considering in the characterization of the brain in terms of its network connectivity. The first is the all-important issue of intersubject diversity. While in invertebrates it is sometimes possible to recognize the same individual neurons across subjects, in mammals it is not even possible to match the same types of neurons bilaterally within subject, such as in motor neurons innervating symmetric muscles [104]. In humans, intersubject variability is already very considerable at the regional level [105] and can be expected to be extraordinary large at the level of individual neurons across subjects.

The second vital element of brain circuitry is structural plasticity, that is, dynamical changes in the synaptic connectivity not just during development but throughout adulthood. In the cortex of normally behaving mice, for example, 4% of axonal boutons change over the course of a few months [106], with similar proportions reported in dendritic spines [107]. Abundant experimental evidence suggests that this form of plasticity is activity- and experience-dependent [108–111]. This is just one of many mechanisms underlying neural plasticity across spatial and temporal scales, from short- or long-term alterations in synaptic strengths to neurogenesis [112], which are believed to support memory storage [113, 114]. 

Much as the brain is in constant flux and its functional connectivity continuously changes with every spike and synaptic discharge, so is the mind never exactly the same before and after instantiating each and every subjective representation. These aspects of both brains and minds are strongly resonant with Heraclitus' “panta rhei” [115].

## 6. Quantifying Declarative Mental States: Semantic Maps

One may find it disturbing that despite knowing so much about the structure and activity of the brain, we have a hard time “guessing” what it is thinking. But it is somehow even more peculiar that in spite of direct, detailed, continuous, and complete access to each and all conscious mental states we experience, we find it difficult to describe them comprehensively, let alone quantitatively. Indeed, it seems absurd that we can measure the concentration of Substance P in single neurons to the fifth significant digit; yet we can only measure the resulting sensation of pain semiqualitatively on a 7-point discrete scale. In order to bring the study of conscious content into the realm of hard science, we need to devise a quantitative measurement system for subjective states [116].

Language has often been considered a convenient proxy to access mental states, if not the most direct tool to describe them. The scientific characterization of the meaning of language, or semantic analysis, has a long history and remains one of the most active research areas in (computational) linguistics. Here we do not aim to review or even to provide a balanced commentary on the state of the art of semantic analysis techniques. Instead, we introduce and explain a very specific, nonconventional approach to this problem that is particularly pertinent to the topic of this spotlight paper. 

Most if not all of the best known computational methods of semantic analysis are based on (variations of) the common principle that the meaning of words relates to the contextual occurrence of their use in language [117]. For example apples, oranges, and grapes tend to be used in similar contexts as reflected by their cooccurrence with similar words in the same sentence (e.g., eat, ripe, juice, and vitamin). Thus, they share similar semantics (they are all types of fruit). The notion that word meaning relates to the (relative) frequency of their cooccurrence is shared by many broadly adopted approaches, including Latent Semantic Indexing [118], Latent Dirichlet Allocation [119], Hyperspace Analogue to Language [120], and many others [121]. In practice, these techniques rely on the identification of statistical patterns of word usage in large-scale text corpora by computational parameter extraction. 

Although the details vary among types of computational semantic analysis, words (or more generally, concepts) are often allocated to a multidimensional abstract space such that the location of each concept reflects its meaning. These spaces are sometime referred to as “semantic maps.” Meaningful semantic dimensions of these spaces can be associated with their geometrical shape, a bit like the location on a globe can be described with the polar and azimuth angles, or the size of a cigar can be described by its length and thickness. Alternatively, meanings can be identified with clusters of words in this space. For instance, all fruit words in the previous example would be located in the same region of the space. By nature of its own principle, latent semantic analysis and its variations generate results that are highly context dependent. In other words, the semantics extracted from a cookbook are typically quite different from those detected in movie reviews or obituaries. In fact, use of nonhomogeneous collections of corpora from different domains typically fails to yield meaningful semantics. Moreover, this general class of methodologies tends to produce a large number of highly specific dimensions.

A rather complementary (and historically precedent) goal of lexical semantics has been to seek the fundamental (or at least context independent) dimensions of word meaning. Perhaps the most seminal study in this sense has been that of Osgood's “semantic differentials” [122]. In that work, subjects were asked to rate a large number of words in various hand-picked dimensions defined by two opposite extremes (e.g., soft-hard, fast-slow, clean-dirty, valuable-worthless, and fair-unfair), using a Likert (discrete) scoring scale. Subsequent analysis identified three principal dimensions that were robust to cultural and geographical differences, namely, evaluation (also known as valence: good-bad), potency (strong-weak), and agency (active-passive). A limitation of these studies and other similar psychometric approaches [123] is that they involve human subjects and arbitrary choices of starting terms. Thus, they are not amenable to automated, high-throughput computational extraction.

Word meaning has of course also been characterized for thousands of years in many languages and cultures through the creation of dictionaries. Here the beginning of modern times can be considered to correspond to the systematic (but again, ultimately arbitrary) classification introduced by Roget's Thesaurus of English words and phrases, now accessible online 160 years after its original publication [124]. Among contemporary efforts, the most comprehensive academic resource is Princeton's WordNet [125]. Researchers in computational linguistics are vigorously pursuing the topic of conceptual ontologies [126]. Yet, it remains to be established if and how formal ontological theories could map semantic spaces such as those generated by latent semantic analyses.

We have recently introduced a novel paradigm for semantic mapping that allows systematic construction of a low-dimensional metric system for the context-independent (“fundamental”) meaning of words [127]. Our approach combines certain elements of Osgood's work (use of antithetical meanings) with the scalable use of “objective” dictionaries. Specifically, using a novel self-organization process, we constructed a semantic map of natural language that simultaneously represents synonymy and antonymy. Synonyms and antonyms are commonly listed in dictionaries for most terms. We extracted these relationships from digitally accessible dictionaries (Microsoft Word and Princeton's WordNet) in each of several languages (English, French, German, and Spanish). For each dictionary and language, we initially allocated words at random locations in a finite, multidimensional spherical space. Then we started moving the position of every word following a simple rule: every word would “attract” its synonyms and “repel” its antonyms. Thus, pairs of synonyms would tend to move closer to each other, and pairs of antonyms would move farther apart (within the bounds of the multidimensional sphere).

This process “converges” in the sense that all words reach a stable position that could not be further improved in terms of proximity to synonyms and distance from antonyms. The resulting space only had a limited number (~4) of statistically significant dimensions. This means that, even if the starting space is a homogeneous sphere of many (~100) dimensions, the resulting emergent shape can be quite completely described with just four numbers. Most importantly, the emergent semantics of the map's principal components are clearly identifiable: the first three correspond to the meanings of “good/bad” (valence), “calm/excited” (arousal), and “open/closed” (freedom), respectively. The semantic map is sufficiently robust to allow the automated extraction of synonyms and antonyms not originally present in the dictionaries used to construct the map, as well as to predict connotation from their coordinates. 

The map's geometric characteristics include a bimodal distribution of the first component, increasing kurtosis of subsequent (unimodal) components, and a U-shaped maximum-spread planar projection. Both the semantic content and the main geometric features of the map are consistent between dictionaries, among tested Western languages, and with previously established psychometric measures. Some of the mathematical formalism and speculative interpretations are elaborated in a second follow-up paper [128]. Interestingly, the main emerging dimensions of this semantic map loosely correspond to the primary modulatory neurotransmitter systems in the mammalian brain [129].

The previous paradigm can be expanded with appropriate adaptations to extract additional, independent dimensions of word meaning by considering other linguistic relations besides synonyms and antonyms. In particular, the relationship “is-a” (hypernyms and hyponyms) captures the abstractness (or more precisely the ontological generality) of words. For example, the statements “Mickey is a mouse”, “the mouse is a rodent”, and “a rodent is an animal” reflect a hierarchy of concepts from the more concrete, or rather specific (Mickey), to the more abstract/general (animal), whereas rodent is a hypernym of mouse and a hyponym of animal. In this paper we refer to this property as “abstractness” because “generality” might be confused to indicate how common a term is (usage frequency).

Is-a relationships are commonly used in dictionary definitions following the classic recipe of Aristotle's Logic “A is a type of B with property C” [130]. However, hypernyms and hyponyms are seldom listed in immediately machine-readable form in digital collections, the way synonyms and antonyms are. One exception is provided once again by WordNet, which explicitly provides is-a relationships among many of its terms. Unlike synonyms and antonyms, which are symmetric relations (if A is synonym of B, B is synonym of A), hypernyms and hyponyms are directional and mutually antisymmetric (if A is hypernym of B, B is hyponym of A). We thus changed the form of the energy functional in the previously described optimization procedure [131].

The resulting allocation of words in space yielded a ranking of all terms along a single dimension, that is, a simple scalar measure of their abstractness (ontological generality). For example, the ten “top” scoring terms (out of ~124,000) are entity, physical entity, psychological feature, auditory communication, unmake, cognition, knowledge, noesis, natural phenomenon, and ability. The bottom (11 ex aequo) of the list reads *Edmontonia*, *Coelophysis*, *Deinocheirus*, *Struthiomimus*, *Deinonychus*, dromaeosaur, *Mononykus olecranus*, oviraptorid, superslasher, *Utahraptor*, and *Velociraptor*.

One advantage of a metric system is that words can be compared in terms of their abstractness even if they are unrelated, for example, the term “governance” (whose abstractness value is 1.726) can be determined to be more abstract than “newspaper” (abstractness of 0.541) even if there are no (sequences of) is-a relationships connecting them. Moreover, because the measure is quantitative, it allows evaluation of relative comparisons. For instance, “power” (1.805) is more abstract than “revolutionary” (1.070). However, the generality of “governance” relative to “newspaper” (abstractness difference:  1.726 − 0.541 = 1.185, abstractness ratio: 1.726/0.541 = 3.190) is greater compared to that of “power” relative to “revolutionary” (abstractness difference: 1.805 − 1.070 = 0.735, abstractness ratio: 1.805/1.070 = 1.687). This opens the possibility to establish a probabilistic estimate of whether a word is more abstract than another.

The metrics of context-independent word meaning along the principal dimensions described previously can be applied to characterize declarative mental states. The most straightforward application is to quantify the content of verbal examples along the main dimensions of the map. This can help in relating semantic content to neural signals. It should be noted that the semantic map described here represents a complementary, rather than alternative, tool to more established latent semantic analyses. While maps produced by the latter are corpus (and context) dependent, this space adds general dimensions that are applicable to all corpora and context. For example, we successfully used the map to rank online collections of movie reviews and biomedical abstract based on the average measure of their words (the very primitive “bag of words” approach) along our dimensions. We indeed found that the first dimension (good-bad) was an excellent quantitative predictor not only of the movie critique score but also (together with the second dimension) of its genre (high valence and arousal: action, low valence and high arousal: drama, high valence and low arousal: romantic comedy, and low valence and arousal: documentaries).

It is also possible to extend the same approach to the has-a relationship (holonyms/meronyms), which is also explicitly included in WordNet. For example, in the sentence “a mouse has a whisker,” the mouse is holonym of whisker and whisker is meronym of mouse. This relationship is (like is-a) also antisymmetric, and we thus expect to be able to extract a “partonomy” scale much with the same approach described previously.

## 7. A Radical View of Reality, Information, Consciousness, and Remaining Challenges

Semantic mapping provides a possible approach to quantifying mental states that can be expressed declaratively. Mental states, or more practically words, sentences, paragraphs, and text in general are allocated in a multidimensional space such that their distances and relative positions reflect particular semantic relationships, including hypernymy/hyponymy (is-a), holonymy/meronymy (has-a), synonymy/antonymy (is similar/opposite to), and in principle many others, such as causation and cooccurrence [132]. In this framework, mental states and their relationships can be themselves represented as graphs of nodes and edges, respectively. If one believes that (at least some) mental states reflect properties of outer reality, it is possible to conceive reality itself as occurring in a giant graph in which any possible observable is a node, and edges correspond to probabilities that two observables would cooccur. We call this conceptual construct the Universal Reality Graph.

In this view, reality would unfold in time as a sequence of events constituting patterns of activation of subsets of nodes and all edges among them within the University Reality Graph. Any agent capable of observation will witness a subset of these activation patterns, that is, a sequence of partial events, each consisting of a collection of active nodes and edges. At each moment of experience, every agent would learn some (but not all) of those associations s/he witnesses. In this process, the agent would progressively form a mental graph representing part of his/her experienced history, which is itself part of the general occurrence of reality, sampled at each instant from the Universal Reality Graph.

While this notion of reality as a graph is purely speculative, even more extreme theories have proposed that physical reality is ultimately a product of information (“It from Bit”), rather than the other way around [133]. Most importantly, the possibility to conceive reality as a graph offers interesting vistas on the solution of the mind-brain problem. If agents form graph-like minds to represent (and therefore predict) their experience of graph-like reality, it stands to reason that the fittest physical substrates selected by evolution to encode these representations be themselves graph like, namely, brain networks. *The relationship between minds and brains could then be resolved as a mapping between their respective graphs and their embeddings*. 

In this framework, the fundamental operation to grow a mind is pairwise association between observables [134], that is, establishment of edges between nodes in the mental graph based on corresponding experiences in the reality graph. An interesting aspect of mental representation is that we only learn a small fraction of associations from those observed in reality. In particular, our ability to learn is gated by previously acquired background information. We have recently proposed that this constraint may be a consequence of the spatial relationship among the tree-like shaped neuronal axons and dendrites that underlie brain connectivity [135, 136]. Specifically, in order for new synapses to be formed, the axon of the presynaptic neuron must be sufficiently close to a dendrite of the postsynaptic neuron, arguably because of preexisting connectivity with other neurons encoding for related knowledge.

These ideas are also consonant with the Information Integration Theory (IIT) of consciousness [137], which is emerging as a leading candidate among the fundamental theories of mental content. The underlying assumption of IIT is that consciousness is fundamentally a property of information processing. Specifically, according to the IIT, when a brain (or in principle any other computing device) is in a particular state, its amount of consciousness, called Phi, depends not only on the actual content represented in that state but also on the absence of all contents represented in the states that are not being (but could be) instantiated. Thus silent neurons contribute to the conscious state as much as the active neurons, because consciousness depends as much on the content that *could* be represented by the network as on the content that is actually being represented. Therefore, consciousness is a product of the integrated activity in the network and is measured by information integration, a property that has been defined in graph structures [138].

While the IIT profoundly links consciousness to information [139], its cognitive underpinning is shared by other theories (e.g., [140]) and experimental approaches [141]. A crucial and unique outcome of IIT, however, is that the definition of integrated information enables a geometric characterization of mental states or qualia [142]. This can in principle provide a neurally based bottom-up correlate to the spaces that emerge from top-down semantic mapping of natural language. If the information processing product of neural network activity can be shown to correspond mathematically to a quantitative description of subjective mental content, the brain-mind problem would be effectively resolved.

Information is not only an essential element of consciousness, reality, and brain activity, but also of communication among conscious agents. Consider a dialogue between two individuals, in which one tells the other: “It's almost midnight, I'm tired. I had such a day.” What does that *mean*? More precisely, what does it mean to the speaking individual, and what does it mean to the listener? Shannon's notion of information [143] captures the reduction of uncertainty of the listener's mind upon receiving the message. Assuming that the second individual had no idea of what time it was, whether the first person was rested or fatigued, and so forth, communication is indeed informative. Mapping brain and mental states, however, opens another perspective on the meaning of communication. Consider the brain/mind state X of the first individual (being tired, etc.), and suppose that the goal of the message was to instantiate X in the second individual. As a result of communication, however, the second individual's brain/mental state is Y rather than X. If Y equaled X, communication would be 100% perfect, but that is never the case.

The listener's understanding of “it's almost midnight” may be fairly close to the speaker's meaning of those words, but even in that case, “midnight” could be associated with different feelings and memories in the two individuals, and the term “almost” might be interpreted as 20 minutes by the first individual and as 2 minutes by the second. When we analyze the second portion of the message, “I'm tired”, we realize that the listener will think of his/her notion of tiredness, which is at best a coarse approximation of the actual state expressed by the speaker, for example, in terms of physical versus mental, chronic versus acute, concerned versus conversational, and so forth. The connotation of “I had such a day” is even more prone to subjective interpretation. The listener may think of his/her days s/he would describe as “such a day”, but those days and associated sentiments are likely quite different from the events and related mental state the speaker was referring to.

This simple example can be generalized to all of human communications. How much of the intended meaning is effectively transmitted between communicating conscious individuals on average? Even in the most favorable cases of colleagues discussing their joint work or spouses talking about their family, we speculate that communication hovers between 50% and 85%. If this is the case, the mean human communication between any two individuals (including casual interactions) might be 10% or less. Even reminiscence and planning (retrieval of autobiographic and prospective memories, resp.), as well as other forms of internal dialogue, can be viewed as special cases of communication (with oneself). Such a type of communication between two instances of the same individual at different points in time can be expected to be much more effective than between different human beings, but even in those situations it will not be perfectly effective, as the mind is in constant flux. In all cases, mental state quantification by semantic mapping and its corresponding neural correlates in brain activity spaces could dramatically enhance communication effectiveness, deeply altering human relationship.

## 8. Concluding Remarks: A Vision Forward

The relationship between mind and matter has perhaps been, in one form or another, the most debated issue in the history of human thought, and it still constitutes, in the modern “mind-brain” incarnation, an open scientific and philosophical problem. Specifically, why do certain brain states “feel” like something, and why specific brain states feel the way they do? We have proposed that a satisfactory answer can ultimately come from mathematics, if the abstract spaces of brain activity and mental content can be quantitatively characterized and geometrically mapped onto each other. Such a “solution” will connect the conscious mind and the relevant aspect of brain states by demonstrating the equivalence of their properties, much like statistical mechanics and thermodynamics are nowadays accepted as one and the same phenomenon, even if they are practically treated as distinct for every day purposes.

We argued that semantic maps constitute a useful initial framework to establish a rigorous description of the mind and that network connectivity provides the most informative constraint on brain dynamics. However, defining the proper mathematical states to effectively bridge brain and mind still constitutes a formidable challenge. State-of-the-art semantic maps only scratch the surface of the necessary quantification of the human mind. Next-generation voice recognition and optical character recognition software programs might soon enable real-time acquisition and analysis of the complete life-long natural language corpus experienced by an individual. Exhaustive compendia of semantic relationships could be extracted from such a resource, enabling the creation of a comprehensive semantic map for that individual. Such a resource could then be used to systematically report subjective mental states. 

While the main obstacle in quantifying mental content appears to be the required paradigm shift towards a science of first-person perspective, neuroscience faces mostly a technological hurdle in creating brain-wide neuron-level maps of network connectivity and activity. Specifically, existing techniques can indeed map all of synaptic circuitries, but only in a very small volume (a fraction of cubic millimeter) of nervous tissue [144], only in animal models, and not *in vivo*. Other techniques to analyze neuronal anatomy, only hinting at the *potential* connectivity [75, 145], are possibly scalable to entire brains of live animals [146, 147], but again not human beings, let alone in normal behavioral conditions. The only noninvasive imaging techniques available to investigate the human brain (e.g., [148]) are by many orders of magnitude too coarse to probe the level of neurons and spike.

Several “futuristic” scenarios have been proposed to solve the technological gap between small-scale animal-model neuron-level analysis and a full activity map of all neurons and synapses of a sentient human brain [149], such as the eventual adoption of nanotechnology [150]. One present-day partial solution is to use molecular homology to identify existing correspondences between neuron types in rodents and humans by comprehensive genetic mapping [151] and single-neuron sequencing [152]. The subsequent extension of rodent brain connectivity to human cognitive architecture would only be tentative, requiring extensive computational testing and refinement by multiscale simulation [153]. An initial pilot project in this regard might tackle a suitable brain region (and related computational functions), such as the mammalian hippocampus [154].

Assuming that, at least in principle, technological advancements enabled accumulation of sufficient datasets to adequately map the neuronal activity of the human brain, such a feat would likely involve massive automation. High-throughput, machine-acquired, and large-scale data poses the outstanding matter of human interpretation [155–157]. This issue has recently promoted considerable growth in the field of neuroinformatics [158], that is, the establishment of an information framework for neuroscience (e.g., databases and other electronic resources), which is especially needed in computational neuroanatomy [159]. Recent initiatives have proposed a formalism to represent connectivity structure in neuronal network models [160] and seeded web-based multimodal connectivity databases [161]. A parallel informatics effort is required to enable storage, manipulation, and analysis of machine- and human-readable empirical data on cognitive functions, behaviors, and introspection [162, 163]. The neuroinformatics of language might provide a useful bridge between neural and cognitive frameworks [164].

The proposed vision offers a path towards a mathematical solution of the relationship between brain and mind that is consistent with contemporary philosophical positions [165]. Tremendous advancements in physics, chemistry, and biology provided an increasingly unified understanding of the material world. Bridging neuroscience with a quantitative description of inner subjective life may provide a fundamental closure to human scientific inquiry.
